# Multifocal Renal Cell Carcinoma: Clinicopathologic Features and Outcomes for Tumors **≤**4 cm

**DOI:** 10.1155/2008/518091

**Published:** 2008-07-15

**Authors:** Paul L. Crispen, Christine M. Lohse, Michael L. Blute

**Affiliations:** ^1^Departments of Urology, Mayo Clinic, Rochester, MN 55905, USA; ^2^Department of Health Sciences Research, Mayo Clinic, Rochester, MN 55905, USA

## Abstract

A significant increase in the incidental detection of small renal tumors has been observed with the routine use of cross-sectional abdominal imaging. However, the proportion of small renal tumors associated with multifocal RCC has yet to be established. Here then, we report our experience with the treatment of multifocal RCC in which the primary tumor was ≤4 cm. In our series of 1113 RCC patients, 5.4% (60/1113) had multifocal disease at the time of nephrectomy. Discordant histology was present in 17% (10/60) of patients with multifocal RCC. Nephron sparing surgery was utilized more frequently in patients with solitary tumors. Overall, cancer-specific, and distant metastasis-free survival appeared to be similar between multifocal and solitary tumors. These findings are consistent with previous series which evaluated multifocal RCC with tumors >4 cm. With the known incidence of multifocality RCC, careful inspection of the entire renal unit should be performed when performing nephron sparing surgery.

## 1. INTRODUCTION

The routine use of cross-sectional abdominal imaging has led to a significant increase in the diagnosis of renal cell carcinoma (RCC) [1, 2]. An estimated 51000 new cases of cancer of the kidney and renal pelvis were diagnosed in 2007, with the vast majority representing RCC [3]. While the preponderance of patients with sporadic RCC will have solitary tumors, 4–20% of patients will have multifocal RCC at the time of diagnosis [4–9]. This is in contrast to patients with hereditary forms of RCC, such as von Hippel-Lindau, Birt-Hogg-Dubé, and hereditary papillary renal carcinoma, who typically have multifocal disease at the time of presentation [10–12]. Whether a patient with multifocal RCC has sporadic or hereditary disease, treatment decisions are based on balancing the preservation of renal function with oncologic efficacy. This is especially true in the case of small (≤4 cm) renal tumors, which are often amenable to nephron sparing surgery (NSS) [13, 14].

Although several previous series have reported on the incidence of multifocality (Table 1), there are limited data on the incidence and outcomes of patients with small (≤4 cm) sporadic multifocal RCC. Here then, we review our experience with the management and outcomes of patients with multifocal sporadic RCC in which the primary tumor size was ≤4 cm.

## 2. MATERIALS AND METHODS

We studied 1113 patients treated with radical nephrectomy or NSS for sporadic, pNX/pN0, pM0 RCC ≤4.0 cm between 1970 and 2004. Of these, 1053 (94.6%) patients had a solitary, unilateral RCC. The remaining 60 (5.4%) patients had unilateral multifocal RCC. Patients with bilateral disease at time of presentation were excluded from analysis. Clinical and pathologic variables were compared between patients with multifocal and solitary tumors. Clinical variables evaluated included patient age, gender, symptoms at presentation, ECOG performance status, and type of surgery. Pathologic features evaluated included 2002 primary tumor classification, histologic subtype, nuclear grade, presence of histologic necrosis, and sarcomatoid differentiation. Histologic subtype was assigned following the recommendations of the 1997 Union Internationale Contre le Cancer and American Joint Committee on Cancer workshop on the classification of RCC [15]. For the 60 patients with multifocal RCC, pathologic characteristics of the largest tumor were summarized, with the exception of histologic subtype. All pathologic specimens were reviewed by a single urologic pathologist. Patient followup data are obtained and maintained through our nephrectomy registry. Information on patients who do not follow up at our institution is obtained by a registered nurse via outside medical records, patient/physician correspondence, or death certificates. Fewer than 3% of the patients in the Nephrectomy Registry have been lost to follow up.

Clinical and pathologic features between the multifocal and solitary groups were compared using Wilcoxon rank sum, chi-square, and Fisher's exact tests. Overall, cancer-specific and distant metastases-free survivals were estimated using the Kaplan-Meier method and overall survival was compared between patient groups using the log-rank test. All tests were two-sided and *P*-values less than .05 were considered statistically significant. Statistical analysis was performed using SAS software (SAS Institute, Cary, NC, USA).

## 3. RESULTS

Multifocal RCC was present in 5.4% (60/1113) of patients with a primary tumor ≤ 4 cm. Clinical and pathologic features between patients with solitary and multifocal RCC tumors ≤ 4.0 cm are summarized in Table 2. Multifocal disease was suspected in only 27% (16/60) of patients based on preoperative imaging. Median age at surgery for the solitary patients was 64 years (mean 62.3; range 22–87) compared with 67.5 years (mean 64.1; range 21–82) for the multifocal patients (*P* = .147). Median tumor size for the solitary patients was 3.0 cm (mean 2.8; range 0.2–4.0) compared with 2.9 cm (mean 2.8; range 0.4–4.0) for the multifocal patients (*P* = .631). A comparison of histologic subtype is shown in Table 3. Note that 10/60 (17%) of the patients with multifocal RCC had multiple tumors of different histologic subtypes.

Among the 1053 patients with solitary RCC, 414 died at a median of 7.7 years following surgery (range 1 day to 37.0 years), including 29 who died from RCC at a median of 5.5 years following surgery (range 0.5–21.5). Among the 639 patients who were still alive at last followup, the median duration of followup was 6.8 years (range 2 days to 35.1 years). Forty-four patients experienced distant metastases at a median of 4.0 years following surgery (range 0.2–21.5). Sixteen patients experienced a contralateral recurrence at a median of 2.9 years following surgery (range 0.3–13.8).

Among the 60 patients with multifocal RCC, 23 died at a median of 6.1 years following surgery (range 0.7–14.1), including 4 who died from RCC at 1.0, 3.4, 5.7, and 9.5 years following surgery, respectively. Among the 37 patients who were still alive at last followup, the median duration of followup was 7.6 years (range 0.7–29.3). Three patients experienced distant metastases at 0.8, 0.9 and 6.8 years following surgery, respectively. Eight patients experienced a contralateral recurrence at a median of 5.5 years following surgery (range 0.6–8.1).

**Figure 1:**
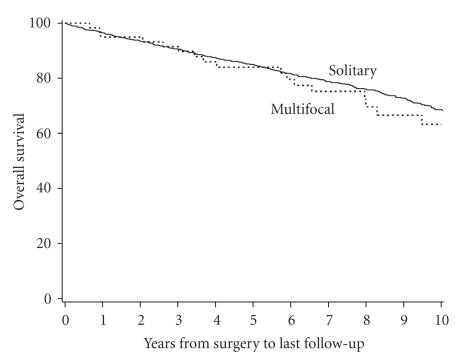
Overall survival in patients with multifocal versus solitary RCC.

Overall survival rates (SE, number still at risk) at 5 and 10 years following surgery were 84.8% (1.2%, 691) and 68.4% (1.7%, 373), respectively, for patients with solitary RCC compared with 84.0% (4.9%, 40) and 63.3% (7.5%, 17), respectively, for patients with multifocal RCC (*P* = .531; Figure 1). Median overall survival for the two groups was 15.2 and 12.3 years, respectively.

**Figure 2:**
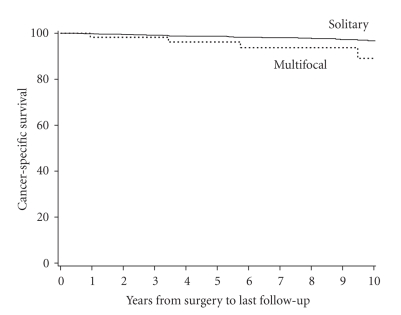
Cancer-specific survival in patients with multifocal versus solitary RCC.

Cancer-specific survival rates (SE, number still at risk) at 5 and 10 years following surgery were 98.7% (0.4%, 691) and 96.7% (0.7%, 373), respectively, for patients with solitary RCC compared with 96.2% (2.6%, 40) and 89.0% (5.7%, 17), respectively, for patients with multifocal RCC (Figure 2). Median cancer-specific survival was not attained for either group during the observed duration of followup. Because so few patients with multifocal RCC died from RCC, no statistical comparison of outcome between the two patient groups was performed.

**Figure 3:**
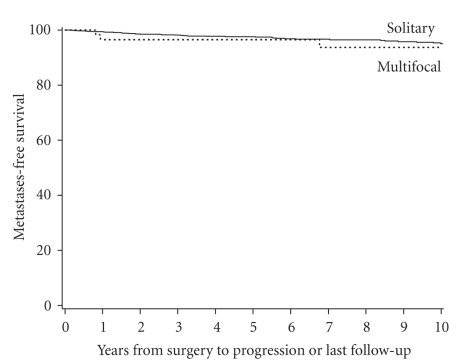
Distant metastasis-free survival in patients with multifocal versus solitary RCC.

Distant metastases-free survival rates (SE, number still at risk) at 5 and 10 years following surgery were 97.6% (0.5%, 687) and 95.1% (0.9%, 368), respectively, for patients with solitary RCC compared with 96.5% (2.1%, 39) and 93.7% (3.7%, 17), respectively, for patients with multifocal RCC (Figure 3). Median distant metastases-free survival was not attained for either group during the observed duration of followup. Because so few patients with multifocal RCC experienced distant metastases, no statistical comparison of outcome between the two patient groups was performed.

**Figure 4:**
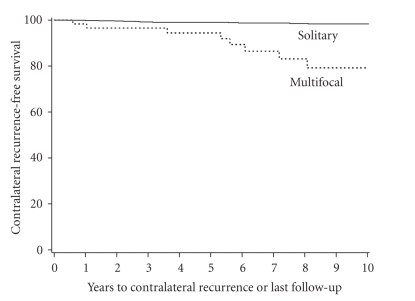
Contralateral recurrence-free survival in patients with multifocal versus solitary RCC.

Contralateral recurrence-free survival rates (SE, number still at risk) at 5 and 10 years following surgery were 99.1% (0.3%, 684) and 98.3% (0.5%, 366), respectively, for patients with solitary RCC compared with 94.4% (3.2%, 38) and 79.2% (6.9%, 16), respectively, for patients with multifocal RCC (*P* < .001; Figure 4). Median contralateral recurrence-free survival was not attained for either group during the observed duration of followup.

## 4. DISCUSSION

In the current series of tumors ≤ 4 cm, the rate of multifocal RCC was similar to prior reports. In a review of series published between 1988 to 1999, multifocal disease was noted in 15.2% (179/1,180) of patients, with 9–100% of the primary tumors being ≤ 4 cm in individual reports [13]. Contemporary series have shown a similar rate of multifocal RCC, ranging from 4.3% to 21.4% [4–9, 17, 18]. With the known incidence of multifocal disease, the ability to identify multifocal renal tumors preoperatively is extremely important and has been evaluated by several series. Kletscher et al. noted that preoperative imaging suggested multifocality in only 44% (7/16) of patients prior to nephrectomy [19]. While in the series by Richstone et al. only 33% of multifocal tumors were identified on preoperative imaging, resulting in the discovery of occult multifocal disease in 3.5% of all patients overall at the time of nephrectomy [7]. Another series by Schlichter et al. investigated the ability of ultrasound and computed tomography to identify multifocality. Upon pathologic evaluation 17.1% (48/281) of radical nephrectomy specimens contained multifocal RCC. However, preoperative imaging was only able to identify 23% (11/48) of multifocal tumors. Collectively, these and the current series demonstrate that preoperative imaging is not a sensitive means of identifying multifocal disease preoperatively. Thus complete mobilization and inspection on the entire kidney is warranted when performing NSS to properly evaluate the presence of multifocal disease.

Several associations have been suggested between clinicopathologic features and the presence of multifocal RCC including primary tumor size, histologic subtype, bilateral disease, nodal status, and tumor stage. However, only two series have performed multivariate analysis when evaluating the associations between multifocality and clinicopathologic features. Baltaci et al. evaluated 103 cases of RCC and noted the incidence of multifocal RCC to be 21.4% [18]. Univariate and multiple logistic regression analysis demonstrated that primary tumor stage was the only independent predictor of multifocality. In the series by Richstone et al. of 1071 radical nephrectomy specimens, 5.3% of patients were noted to have multifocal RCC [7]. Multivariate analysis of this population revealed significant associations between multifocality with papillary subtype, lymph node metastasis, advanced tumor stage (pT4), and bilateral disease. Interestingly, neither series noted a significant association with tumor size and multifocal RCC. This is important to consider when treating small renal tumors, as size alone has not been shown to predict the presence of multifocal disease.

Discordant pathology between the primary and satellite tumors occurs in up to 6–30% of multifocal tumors [4, 7, 19]. A similar rate of discordant histology between the primary and satellite tumors was noted in the current series at 17%. Although it is obvious that separate events are likely responsible for multifocal RCC with discordant histology, the origin of multifocal RCC with concordant histology is not as apparent. However, the evaluation of genetic markers has provided insight into the origin of multifocal RCC. An initial report by Miyake et al. evaluated the loss of heterozygosity (LOH) using 18 satellite markers in 10 patients with multifocal clear cell RCC (ccRCC) [20]. Identical LOH patterns were noted in 80% (8/10) cases, suggesting that multifocal ccRCC represent intrarenal metastasis. In a second report examining the genetic clonality of multifocal ccRCC by Junker et al. 89% (17/19) cases demonstrated identical LOH patterns [17]. In contrast to ccRCC, multifocal papillary RCC appears to represent independent primary tumors. In a report by Jones et al. LOH was examined in 21 patients with multifocal papillary RCC [21]. The majority, 95% (20/21), of cases demonstrated distinct LOH patterns between tumors suggesting that multifocal papillary tumors do not represent intrarenal metastasis, unlike ccRCC.

Survival outcomes following the treatment of multifocal RCC have been evaluated in several series (Table 4). Dimarco et al. reviewed 2373 patients treated for RCC over 30 years. Multifocal disease was present in 4.3% (101/2373) of all patients. Of the patients with multifocal disease 70% (71/101) had multifocal lesions of the same histologic subtype; these patients were utilized to evaluate survival outcomes. Ipsilateral recurrence rates were similar between multifocal and solitary RCC following radical nephrectomy. Contralateral recurrence was more common in patients with multifocal ccRCC with an increased risk ratio of 2.91; however, this increase did not reach statistical significance (*P* = 1.42). However, in a separate report by Bani-Hani et al. a significant association between the risk of contralateral recurrence and multifocality was demonstrated [22]. The association between contralateral recurrence and multifocality was again noticed in current series which only includes RCC ≤4 cm. Cancer-specific survival was similar between patients with multifocal RCC at 1, 5, and 10 years following nephrectomy in patients with clear cell and papillary RCC [23]. Similar findings were noted by Lang et al. in the review of 255 patients undergoing radical nephrectomy [5]. In this series multifocality was present in 14.5% (37/255) of patients undergoing radical nephrectomy for RCC. Multifocality was not associated with metastatic progression, cancer-specific or overall survival in patients treated with radical nephrectomy during median followup of 183 months compared to patients treated for solitary tumors. Additionally, in the report by Richstone et al. no significant difference was noted in 5 year disease-free (71.5% versus 73.2%) and overall (75.2% versus 79.3%) survival when comparing patients with multifocal and solitary RCC [7]. In another study by Méjean et al. focusing on papillary RCC, the presence of multifocal disease was not a significant predictor of overall survival compared to solitary tumors [16]. Collectively these results, with the inclusion of the results from the current series, suggest that cancer specific outcomes are equivalent between patients with multifocal and solitary RCC when treated with radical nephrectomy.

NSS in the management of solitary RCC provides equivalent oncologic efficacy while improving overall survival compared to radical nephrectomy [13, 24, 25]. Although there are limited data on the efficacy of NSS when treating sporadic multifocal RCC, available data suggest that NSS has equivalent oncologic efficacy when treating multifocal disease. An initial report from the Mayo Clinic by Blute et al. reviewed 16 cases of multifocal tumors treated with NSS [23]. 6/16 (38%) of these patients had a solitary kidney at the time of presentation. Local recurrence was noted in 2/16 patients at 1.7 and 2.8 years following NSS. Recurrent disease was treated with repeat NSS in one patient and systemic therapy in the other. Cancer specific survival was 100% at 5 years, however 2/16 patients died of RCC at 6 and 11 years postoperatively. Because of the small number of patients treated, survival outcome comparisons were not made between patients treated with NSS and radical nephrectomy.

Additionally, when considering disease recurrence in patients undergoing NSS for multifocal disease, it can be difficult discriminating recurrent and persistent disease. Local treatment failures in patients previously treated for multifocal RCC does not automatically indicate radical nephrectomy of the renal remnant. Two recent series have reported the feasibility and outcomes of salvage partial nephrectomy in patients with local recurrence following a previous partial nephrectomy [26, 27]. Bratslavsky et al. reported on 11 patients undergoing salvage partial nephrectomies for von Hippel-Lindau disease [27]. Three renal remnants were lost while attempting to preserve renal function, and 46% of cases were associated with major postoperative complications. It should be noted that salvage partial nephrectomy in this series was defined as at least the third partial nephrectomy on the renal remnant. A second series by Magera et al. reported outcomes following salvage partial nephrectomy in 18 patients (8 solitary kidneys, 7 patients with von Hippel-Lindau disease) [26]. Postoperative complications were noted in 28% of patients. Although there was no reported loss of a renal remnant in this series, chronic renal insufficiency (serum creatinine > 2.0 mg/dl) was noted in one patient and chronic renal failure (serum creatine > 2.5 mg/dl) in two others. Obviously, salvage partial nephrectomy was performed for absolute indications in all cases in an attempt to preserve renal function and avoid long-term hemodialysis.

 Additional data from series evaluating the efficacy of NSS for multifocal RCC in patients with von Hippel-Lindau disease have demonstrated the significant impact of tumor size on future disease progression. In the series by Duffey et al., NSS was utilized in 97% of patients with tumors ≤3 cm compared to 69% in patients with tumors >3 cm [14]. Progression to metastatic disease was noted in 27% of patients treated for tumors >3 cm (mean followup 73 months); however, no patients treated for tumors ≤3 cm demonstrated disease progression (mean followup 58 months). Although these data suggest that small multifocal RCCs can be treated with NSS, with a low rate of progression to metastatic disease, direct comparisons between the natural history of sporadic and hereditary multifocal RCC should be made with caution.

## 5. CONCLUSIONS

In the current series multifocal RCC was present in 5.4% of patients with tumors ≤4 cm. Multifocal RCC presents several challenges in terms of diagnosis and treatment. Although multifocal disease is present in only a small proportion of patients with RCC, recognition of multifocality is important to ensure appropriate treatment. As preoperative imaging is an imperfect means of establishing the presence of multifocal disease, careful intraoperative inspection of the entire renal unit should be performed routinely during NSS. Based on the current and other available series, the presence of multifocal disease does not portend a worse prognosis compared to solitary RCC. Additional evaluation of the role of NSS in patients with multifocal sporadic RCC, especially among those with tumors ≤4 cm, is warranted.

## Figures and Tables

**Table 1:** Incidence of multifocal RCC in prior series. ccRCC = clear cell renal cell carcinoma; pRCC = papillary renal cell carcinoma; NA = not available.

Author	Year	Total Patients	Multifocal (%)	Median Tumor	≤4 cm (%)
				Size (cm)	
Richstone	2004	1071	57 (5.3)	5.0	16%
Dimarco	2004	2373	101 (4.3)	4.5 ccRCC	NA
				4.0 pRCC	
Lang	2004	255	37 (14.5)	NA	12.9%
Junker	2002	372	61 (16.4)	NA	NA
Karayiannis	2002	56	10 (17.8)	7.5	30%
Schlichter	2000	281	48 (17.1)	NA	NA
Baltaci	2000	103	22 (21.4)	7.5	24.1%
Wunderlich	1999	260	36 (13.9)	NA	NA

**Table 2:** Clinical and pathologic features.

	Solitary *N* = 1053	Multifocal *N* = 60	
Feature	N (%)		*P*-value
Age at Surgery (years)			
* * * *<65	554 (52.6)	23 (38.3)	.031
* * * *≥65	499 (47.4)	37 (61.7)	
Sex			
* * * *Female	339 (32.2)	11 (18.3)	.025
* * * *Male	714 (67.8)	49 (81.7)	
Symptoms at presentation			
* * * *Absent	582 (55.3)	39 (65.0)	.140
* * * *Present	471 (44.7)	21 (35.0)	
Constitutional symptoms at presentation			
* * * *Absent	893 (84.8)	49 (81.7)	.512
* * * *Present	160 (15.2)	11 (18.3)	
ECOG Performance status (*N* = 903)			
* * * *0	749 (88.4)	50 (89.3)	.846
* * * *≥1	98 (11.6)	6 (10.7)	
Type of Surgery			
* * * *Open radical nephrectomy	532 (50.5)	41 (68.3)	.007
* * * *Open nephron-sparing surgery	460 (43.7)	16 (26.7)	
* * * *Laparoscopic radical nephrectomy	23 (2.2)	3 (5.0)	
* * * *Laparoscopic nephron-sparing surgery	38 (3.6)	0	
2002 Primary tumor classification			
* * * *pT1a	1020 (96.9)	59 (98.3)	1.00
* * * *pT3a	20 (1.9)	1 (1.7)	
* * * *pT3b	11 (1.0)	0	
* * * *pT3c	2 (0.2)	0	
RCC Nuclear grade			
* * * *1	147 (14.0)	6 (10.0)	.672
* * * *2	673 (63.9)	40 (66.7)	
* * * *3	221 (21.0)	14 (23.3)	
* * * *4	12 (1.1)	0	
Coagulative tumor necrosis			
* * * *Absent	921 (87.5)	54 (90.0)	.562
* * * *Present	132 (12.5)	6 (10.0)	
Sarcomatoid Differentiation			
* * * *Absent	1048 (99.5)	60 (100.0)	1.00
* * * *Present	5 (0.5)	0	

**Table 3:** RCC histologic subtype.

Patient Group	N (%)
Solitary RCC (*N* = 1053)	
* * * *Clear cell	771 (73.2)
* * * *Papillary	226 (21.5)
* * * *Chromophobe	45 (4.3)
* * * *Collecting duct	2 (0.2)
* * * *RCC, not otherwise specified	9 (0.9)
Multifocal RCC (*N* = 60)	
* * * *Clear cell	26 (43.3)
* * * *Papillary	23 (38.3)
* * * *Chromophobe	1 (1.7)
* * * *Clear cell + papillary	8 (13.3)
* * * *Clear cell + chromophobe	1 (1.7)
* * * *Papillary + chromophobe	1 (1.7)

**Table 4:** Cancer-specific survival in patients with multifocal versus solitary RCC. ccRCC = clear cell renal cell carcinoma; pRCC = papillary renal cell carcinoma; NS = not significant.

Author	N multifocal	5 year survival	N solitary	5 year survival	*P*-value
Dimarco et al. [4]	40 (ccRCC)	74.6%	1934 (ccRCC)	69.0%	.47
	29 (pRCC)	100%	237(pRCC)	86.6%	.62
Lang et al. [5]	37	74.0%	218	79.9%	.26
Richstone et al. [7]	51	71.5%*	938	73.2%*	NS
Méjean et al. [16]	28 (pRCC)	96%	30 (pRCC)	100%	.53

*Disease-free survival.
